# The Effects of Prostaglandin E_2_ Treatment on the Secretory Function of Mare Corpus Luteum Depends on the Site of Application: An *in vivo* Study

**DOI:** 10.3389/fvets.2021.753796

**Published:** 2022-02-15

**Authors:** Katarzyna K. Piotrowska-Tomala, Agnieszka W. Jonczyk, Anna Z. Szóstek-Mioduchowska, Ewelina Żebrowska, Graca Ferreira-Dias, Dariusz J. Skarzynski

**Affiliations:** ^1^Department Reproductive Immunology and Pathology, Institute of Animal Reproduction and Food Research, Polish Academy of Sciences, Olsztyn, Poland; ^2^Faculty of Veterinary Medicine, CIISA - Centre for Interdisciplinary Research in Animal Health, University of Lisbon, Lisbon, Portugal

**Keywords:** prostaglandin E_2_, human chorion gonadotropin, corpus luteum, progesterone, mare

## Abstract

We examined the effect of prostaglandin (PG) E_2_ on the secretory function of equine corpus luteum (CL), according to the application site: intra-CL injection vs. an intrauterine (intra-U) administration. Moreover, the effect of intra-CL injection vs. intra-U administration of both luteotropic factors: PGE_2_ and human chorionic gonadotropin (hCG) as a positive control, on CL function was additionally compared. Mares were assigned to the groups (*n* = 6 per group): (1) an intra-CL saline injection (control); (2) an intra-CL injection of PGE_2_ (5 mg/ml); (3) an intra-CL injection of hCG (1,500 IU/ml); (4) an intra-U saline administration (control); (5) an intra-U administration of PGE_2_ (5 mg/5 ml); (6) an intra-U administration of hCG (1,500 IU/5 ml). Progesterone (P_4_) and PGE_2_ concentrations were measured in blood plasma samples collected at −2, −1, and 0 (pre-treatment), and at 1, 2, 3, 4, 6, 8, 10, 12, and 24 h after treatments. Moreover, effects of different doses of PGE_2_ application on the concentration of total PGF_2α_ (PGF_2α_ and its main metabolite 13,14-dihydro-15-keto-prostaglandin F_2α_– PGFM) was determined. The time point of PGE_2_, hCG, or saline administration was defined as hour “0” of the experiment. An intra-CL injection of PGE_2_ increased P_4_ and PGE_2_ concentrations between 3 and 4 h or at 3 and 12 h, respectively (*p* < 0.05). While intra-U administration of PGE_2_ elevated P_4_ concentrations between 8 and 24 h, PGE_2_ was upregulated at 1 h and between 3 and 4 h (*p* < 0.05). An intra-CL injection of hCG increased P_4_ concentrations at 1, 6, and 12 h (*p* < 0.05), while its intra-U administration enhanced P_4_ and PGE_2_ concentrations between 1 and 12 h or at 3 h and between 6 and 10 h, respectively (*p* < 0.05). An application of PGE_2_, dependently on the dose, supports equine CL function, regardless of the application site, consequently leading to differences in both P_4_ and PGE_2_ concentrations in blood plasma.

## Introduction

Corpus luteum (CL) is critical for reproductive cyclicity and pregnancy maintenance, which depends on the supportive action of progesterone (P_4_) secreted by this transient endocrine gland (1–3). The lifespan of CL is controlled by numerous regulatory factors with luteotropic and luteolytic effects (4) such as cytokines, growth factors, P_4_, 17β-estradiol (E_2_), luteinizing hormone (LH), prostaglandin (PG) E_2_, and PGF_2α_, respectively (5–7). Some of these factors are widely applied in veterinary practice for estrus synchronization. Mostly, PGF_2α_ is used for the regulation of the estrous cycles in the mare. However, application of PGE_2_ or LH analogs (human chorionic gonadotropin; hCG and equine chorionic gonadotropin; eCG) are also key areas of veterinarian interests in the control of equine reproduction. In addition, the interesting issues in the veterinary practice are different models of drug administration have been investigated in farm animals (5, 8, 9).

Human chorionic gonadotropin is a glycoprotein purified from the urine of pregnant women (10). This glycoprotein acts as LH, sharing the same receptor (1). The evidence for the presence of the LH/CGR receptor in the reproductive tract of humans and other domestic animals has been previously described (11, 12). Moreover, in mares, the LH receptor is expressed in the endometrium and myometrium during the estrous cycle and anestrus (13). Intramuscular (i.m.) (14), subcutaneous (s.c.) (10), or intravenous (i.v.) (15–17) hCG administration has shown a good efficacy in the induction of ovulation to improve the time of mating in mares. Moreover, in mares at early diestrus, i.m. (18) or i.v. (19) hCG application results in an increase in circulating progestin concentrations. Other studies using hCG found promising results in breeding mares. Intravenous hCG administration has been advocated for use to increase fertility and early equine pregnancy rates (20). In addition, the positive effect of i.v. or i.m. hCG administration on an additional CL formation and an increase in pregnancy rates have been reported in cattle (21–24). Therefore, in our study hCG was used as a control–reference luteotropic factor.

Prostaglandins are key factors in many reproductive processes in mammals, such as luteolysis, fertilization, maternal recognition of pregnancy, and implantation (5). It has been previously demonstrated that PG are produced by the CL in numerous species (25–30). Prostaglandin E_2_ is known as a luteotropic factor (1, 31, 32). Our preliminary *in vitro* study confirmed that PGE_2_ plays a luteotropic role as an auto-paracrine factor stimulating P_4_ production by equine luteal steroidogenic cells and CL tissues (33, 34). The effects of PGE_2_ are mediated by four receptor subtypes, which are encoded by different genes: EP1, EP2, EP3, and EP4 (35). The expression of the EP2 and EP4 receptors in the uterus during the estrous cycle and pregnancy has been reported in mares (36). In contrast to PGE_2_, PGF_2α_ is the main luteolytic agent secreted in pulses from the uterine endometrium of numerous mammals during luteolysis including mares (37–40). Ginther et al. (41) demonstrated that pulses of PGF_2α_ detected before the onset of luteolysis were less frequent per session and less prominent than during and after luteolysis.

According to our *in vitro* studies, in mare, many factors are involved in the secretion of PG from equine CL such as cytokine (42, 43) and from the endometrium such as P_4_, E_2_, oxytocin, LH, or cortisol (44–46) regulating modulating enzymatic cascade of AA metabolism. In the PG production cascade, prostaglandin–endoperoxide synthases (*PTGS2*) convert arachidonic acid (AA) into PGH_2_. The conversion of PGH_2_ into PGF_2α_ and PGE_2_ is catalyzed by PGF_2α_ synthases (*PTGFS*) and PGE_2_ synthases (*PTGES*), respectively. Prostaglandin H_2_ is converted to PGI_2_ by the action of PGI_2_ synthases (*PTGIS*) (47). In addition, PGE_2_ can be converted into PGF_2α_ through PGE2-9-ketoreductase (PGE2-9-K) activation, an enzyme which works also as 20-α-hydroxysteroid dehydrogenase (20α-HSD), converting P_4_ into inactive 20-α-hydroxyprogesterone (20α-OHP) (4, 48). In mares, the aldo-keto reductase (AKR1C23), which has 20α-HSD activity, converting P_4_ to its inactive metabolite, was expressed in the CL (30, 48) and placenta during placentitis (49). Moreover, 15-hydroxyprostaglandin dehydrogenase (PGDH), which is involved in the first step of PG inactivation, leading into the generation of 15-keto-metabolites, was expressed in mares in the CL (50), gravity uterus (51), and presented from 150 days of gestation onwards (52). Similar mechanisms that involved the activity of PGE2-9-K were confirmed in the rabbit ovary (53, 54) and bovine placenta (55, 56). Therefore, due to the analysis of the action of PGE_2_, its conversion into PGF_2α_ should also be considered. The above effect may depend on different interactions between luteotropic PGE_2_ and luteolytic PGF_2α_.

Many studies have discussed the benefits and disadvantages of different routes of PGE_2_ administration and its proper dosages in mares (32, 57, 58). While some studies reported intrafollicular PGE_2_ administration induced ovulation (58), in other studies intrauterine (intra-U) administration of PGE_2_ resulted in prolonged CL (32). Moreover, the positive influence of intracervical administration of PGE_2_ on the preparation of the uterine cervix to parturition in mares has been observed (59).

The area of research seeking the most effective routes and site for administration of luteotropic agents, used for manipulation of the reproductive processes in breeding mares, is still valuable for veterinary practitioners. To the best of our knowledge, no reports have demonstrated so far the action of PGE_2_ on equine mid-luteal CL (day 10 of the estrous cycle) secretory function according to its application site. Therefore, the objective of this study was to determine the effects of PGE_2_ on the secretory function of CL, according to the application site: ultrasound-guided intra-CL injection vs. intra-U administration. Moreover, the effect of intra-CL injection vs. intra-U administration of both luteotropic factors, PGE_2_ and hCG (as a positive control), on CL function was additionally compared. Possibility of the conversion of luteotropic PGE_2_ into luteolytic PGF_2α_ dependently on the dose was also examined.

## Materials and Methods

### Animals and Surgical Procedures

**Figure 1:**
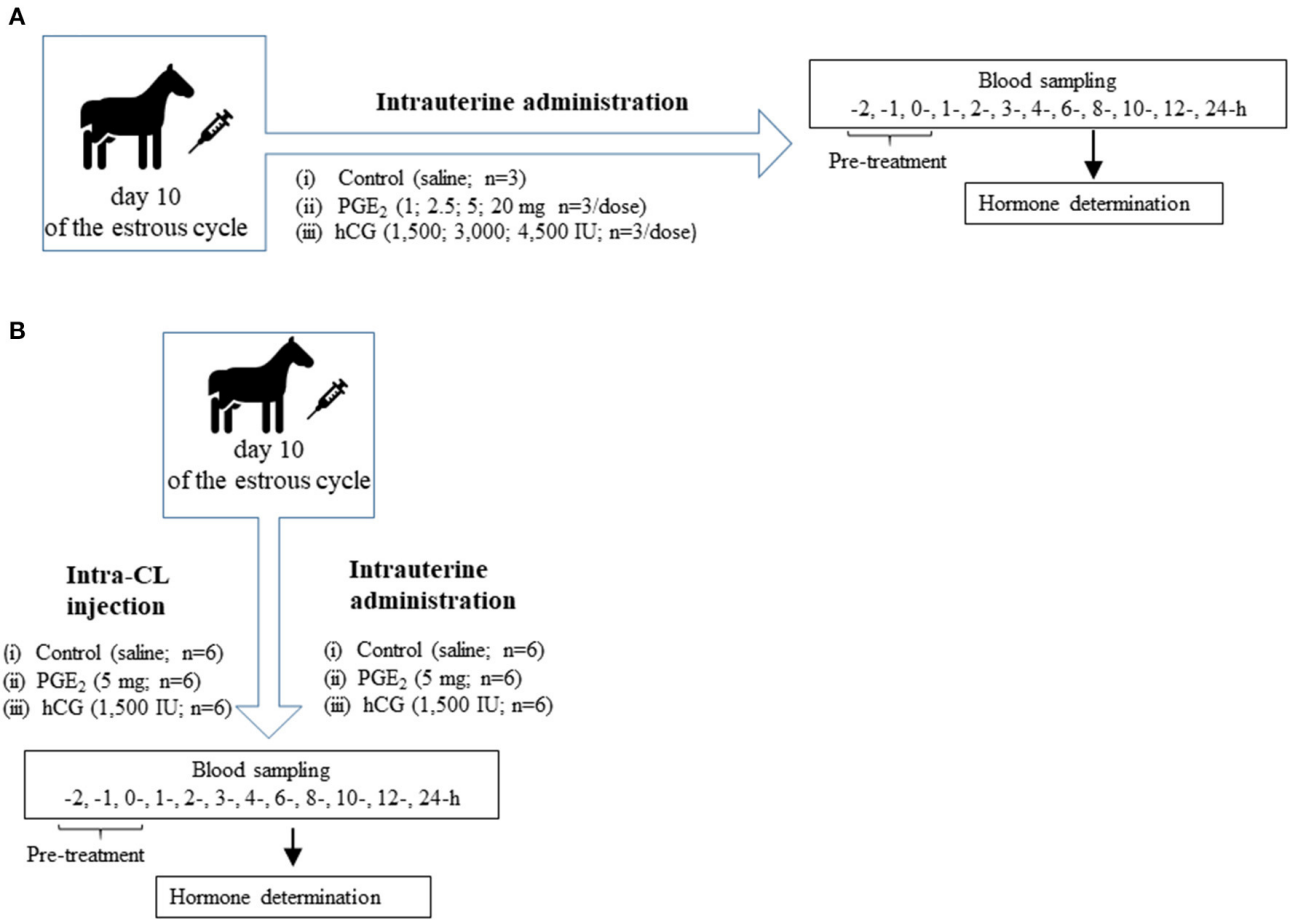
**(A)** Schematic diagram of experiment 1. Mares on day 10 of the estrous cycle were managed as follows: (1) One intrauterine saline administration (control group; *n* = 3); (2) One intrauterine administration of prostaglandin (PG) E_2_ (PGE_2_; 1 mg/5 ml, 2.5 mg/5 ml, 5 mg/5 ml, 20 mg/5 ml; *n* = 3 per dose); (3) One intrauterine administration of human chorionic gonadotropin (hCG; positive control; 1,500 IU/5 ml; 3,000 IU/5 ml, 4,500 IU/5ml; *n* = 3 per dose). After treatment (0 h), blood plasma samples were collected for 24 h throughout the experiment. **(B)** Schematic diagram of experiment 2. Mares on day 10 of the estrous cycle were managed as follows: (1) One intra-CL saline injection (control; *n* = 6); (2) One intra-CL injection of PGE_2_ (5 mg/ml; *n* = 6); (3) One intra-CL injection of hCG (positive, control; 1,500 IU/ml; *n* = 6); (4) One intrauterine saline administration (control group; *n* = 6); (5) One intrauterine administration of PGE_2_ (5 mg/5 ml; *n* = 6); (6) One intrauterine administration of hCG (positive control; 1,500 IU/5 ml; *n* = 6). After treatment (0 h), blood plasma samples were collected for 24 h throughout the experiment.

Fifty-one clinically healthy, non-pregnant, and normally cycling mixed-breed mares (aged 3–13 years, weighing 400 ± 150 kg) were used. The study was conducted between April and September 2016 in Poland. Mares were housed in private stables and were provided *ad libitum* access to water and fed hay and cereal grain. Horses deemed otherwise healthy based on a veterinary physical examination. Animal procedures were conducted in accordance with the EU Directive of the European Parliament and the Council on the protection of animals used for scientific purposes (22 September 2010; no 2010/63/EU), the Polish Parliament Act on Animal Protection (21 August 1997, Journal of Laws 1997 No 111 item 724) with further updates—the Polish Parliament Act on the protection of animals used for scientific or educational purposes (15 January 2015, Journal of Laws 2015 item 266). All animal procedures were designed to avoid or minimize discomfort, distress, and pain to the animals. Procedures were reviewed and accepted following the guidelines of the Local Ethics Committee for Experiments on Animals in Olsztyn, Poland (Approval No. 51/2011). Animals had no abnormalities of the reproductive tract detected by ultrasonic imaging. Prior to the experiment, mares received two doses of a PGF_2α_ analog (5 mg dinoprost, Dinolytic; Zoetis, Poland), 12 days apart, for synchronization of estrus. Follicular development was monitored in mares using transrectal palpation and USG at 12-h intervals during the periovulatory period until ovulation and every 2 days until day 10 (day 0 = day of ovulation). Moreover, structural changes of the CL during the entire estrous cycle were evaluated by ultrasonography with a 7.5-MHz linear probe (MyLabOne Vet Ultrasound System; ESOATE Pie Medica, Genoa, Italy), and visible signs of estrus (i.e., vaginal mucus and standing behavior) were assessed. In addition, the stage of the estrous cycle was confirmed by measurement of peripheral concentrations of P_4_ in blood plasma samples collected from mares. Figure 1 shows the *in vivo* study design where mares (*n* = 51) at day 10 of the estrous cycle were enrolled to the following experiments.

### Intravenous Catheterization

Each mare was sedated with detomidine hydrochloride (Domosedan 0.01 mg/kg i.v.; Orion Pharma Poland Sp, Poland), followed by insertion of a temporary catheter (Intraflon IV cannulae 2.1 × 80 mm 14G, KRUUSE, 121805; KRUSSE Poland) into the jugular vein of mares. Intravenous catheters were flushed with heparinized saline and used for frequent blood sample collections.

### An Intra-CL Injection

Caudal epidural anesthesia was achieved with 4 ml procaine hydrochloride (2% Polocainum Hydrochloricum; Biowet Drwalew, Poland). All intra-CL injections were administered under ultrasound guidance (7.5 MHz linear array transducer, MyLab 30 VET Gold Color Doppler Diagnostic Ultrasound System; ESOATE Pie Medica) through a sterile 1.25 × 50 mm (2-in. 18-gauge) ovum pick-up disposable veterinary injection needle (Bovivet, Poznan, Poland). The transducer and needle guide were coated with a sterile lubricant (Medicum, Lodz, Poland), and positioned within the vagina. The convex transducer was placed in the vagina against the vaginal fornix ipsilateral to the target ovary. The needle was then passed through the vaginal wall, and intraluteal treatments, PGE_2_ (PGE_2_, P0409; Sigma-Aldrich, Saint Louis, Missouri, USA) or hCG (Chorulon; Intervet International B.V., The Netherlands) dissolved in sterile saline solution (1 ml), were injected directly into the CL.

### Intrauterine (Intra-U) Administration

The luteotropic factors were administrated directly into the uterine lumen of mares. The catheter was protected by a sanitary sheath that was broken immediately before the catheter passed through the opening of the cervix. Prostaglandin E_2_ or hCG dissolved in sterile saline solution was infused into the uterine horn using a 5-ml sterile syringe.

### Experimental Design

#### Experiment 1. Dose-Dependent Effect of Prostaglandin E_2_ on CL Function, Compared With Human Chorionic Gonadotropin Action

Experiment 1 design is shown in Figure 1A. The dose-dependent effect of PGE_2_ on blood plasma P_4_ concentrations in mares on day 10 of the estrous cycle was determined as follows: (1) one intra-U saline administration (control group; *n* = 3); (2) one intra-U administration of PGE_2_ (1 mg/5 ml, 2.5 mg/5 ml, 5 mg/5 ml, 20 mg/5 ml; *n* = 3/per dose); (3) one intra-U administration of hCG (positive control; 1,500 IU/5 ml, 3,000 IU/5 ml, 4,500 IU/5 ml; *n* = 3/per dose).

Moreover, the possibility of PGE_2_ conversion into PGF_2α_, dependently on the dose, was also examined. The concentration of total PGF_2α_ (PGF_2α_ plus its main metabolite 13,14-dihydro-15-keto-prostaglandin F_2α_– PGFM) in blood plasma of mares on day 10 of the estrous cycle was determined after different doses of PGE_2_ application (**Table 2**). In mares, PGF_2α_ in the uterine vein reaches systemic circulation and is metabolized in the lungs much via PGDH, resulting in lower concentrations of PGF_2α_ (38, 60). The half-life of PGF_2α_ in mares is short (94 s); therefore, plasma concentrations of PGFM are used to represent changes in PGF_2α_ output (38, 60). The blood sampling was described in Blood Sampling section.

#### Experiment 2. The Comparison of Intra-CL Versus Intra-U Application Site of Prostaglandin E_2_ on CL Function, Compared With Human Chorionic Gonadotropin Action

Experiment 2 design is shown in Figure 1B. To investigate the effect of PGE_2_ according to the application site on the function of equine CL, mares on day 10 of the estrous cycle were managed as follows: (1) an intra-CL saline injection (control; *n* = 6); (2) one intra-CL injection of PGE_2_ (5 mg/ml; *n* = 6); (3) an intra-CL injection of hCG (1,500 IU/ml; *n* = 6); (4) one intra-U saline administration (control; *n* = 6); (5) one intra-U administration of PGE_2_ (5 mg/5 ml; *n* = 6); (6) one intra-U administration of hCG (1,500 IU/5 ml; *n* = 6). Mares (from experiment 1) with intra-U administrations of saline (*n* = 3), PGE_2_ (5 mg/5 ml *n* = 3), and hCG (1,500 IU/5 ml; *n* = 3) were used in experiment 2, respectively. The blood sampling is described in Blood Sampling section.

### Blood Sampling

In mares, blood was aspirated frequently from the jugular vein according to the schedule: at −2, −1, and 0 (pre-treatment), and at 1, 2, 3, 4, 6, 8, 10, 12, and 24 h after injection/administration as shown in Figures 1A,B. The time point of intra-CL injection or intra-U administration of PGE_2_, hCG or saline was defined as hour “0” of the experiment. Blood was aspirated into sterile 10-ml tubes containing 100 μl of 0.3 M EDTA and 1% acetylsalicylic acid, pH 7.4. After centrifugation (2,000 × g for 10 min at 4°C), plasma was stored at −20°C for determination of P_4_, PGE_2_, PGF_2α_, and PGFM concentrations.

### Hormone Determination

Progesterone concentration in blood plasma was measured in duplicates via RIA (P4125 104 I” RIA kit, Immunotech, Czech Republic, IM1188), according to the manufacturer's instructions. The standard curve for P_4_ ranged from 0.1 to 100 ng/ml. The intra- and inter-assay coefficients of variation (CV) were 6.5 and 8.6%, respectively.

Prostaglandin E_2_ was determined in blood samples using commercial ELISA kit (Enzyme Immunoassay kit; Enzo Life Science, Farmingdale, New York, USA, #ADI-901-001), according to the manufacturer's instructions. The standard curve for PGE_2_ ranged from 39.1 to 2,500 pg/ml. The sensitivity of the PGE_2_ assay was 13.4 pg/ml. The cross-reactivity for various prostaglandins and their metabolites was as follows: PGE_2_ 100%, PGE_1_ 70%, PGE_3_ 16.3%, PGF_1α_ 1.4%, PGF_2α_ 0.7%, and 6-keto-PGF_1α_ 0.6%. The intra- and inter-assay CV were 13.1% and 9.7%, respectively. The intra- and inter-assay CV were 5.8 and 5.1%, respectively.

13,14-Dihydro-15-keto-PGF_2α_ (PGFM) was determined in blood samples using a commercial ELISA kit (PGFM Enzyme Competitive ELISA Kit, Invitrogen, Thermo Fisher Scientific, #EIAPGFM, UK), according to the manufacturer's instructions. The standard curve for PGFM ranged from 50 to 3,200 pg/ml. The sensitivity of the PGFM assay was 20.8 pg/ml. The cross-reactivity for various prostaglandins and their metabolites was as follows: PGFM 100%, PGEM 1.5%, PGF_2α_ 0%, and PGE_2_ 0%. The intra- and inter-assay CV were 7.5 and 9.6%, respectively.

Prostaglandin F_2α_ was determined in blood samples using a commercial PGF_2α_ ELISA kit (ENZO Life Sciences Inc., Farmingdale, NY, USA; ADI-901-069) according to the manufacturer's instructions. The standard curve for PGF_2α_ ranged from 3.05 to 50,000 pg/ml. The sensitivity of the PGF_2α_ assay was 6.71 pg/ml. The cross-reactivity for various prostaglandins and their metabolites was as follows: PGF_2α_ 100%, PGF1_α_ 11.82%, PGD_2_ 3.62%, 6-keto-PGF_1α_ 1.38%, PGI_2_ 1.25%, and PGE_2_ 0.77%. The intra- and inter-assay CV were 6.8 and 9.7%, respectively.

### Statistical Analysis

For each statistical analysis, a Gaussian distribution was tested using D'Agostino and Pearson normality test (GraphPad Software version 8.3.0; GraphPad, San Diego, CA, USA). Parametric analyses were performed because normal distribution was assumed. Two-way ANOVA (GraphPad) test was used in experiment 1 (Supplementary Tables 1–3) and in experiment 2 (Supplementary Tables 4, 5). The results were considered significantly different at *p* < 0.05.

**Figure 2:**
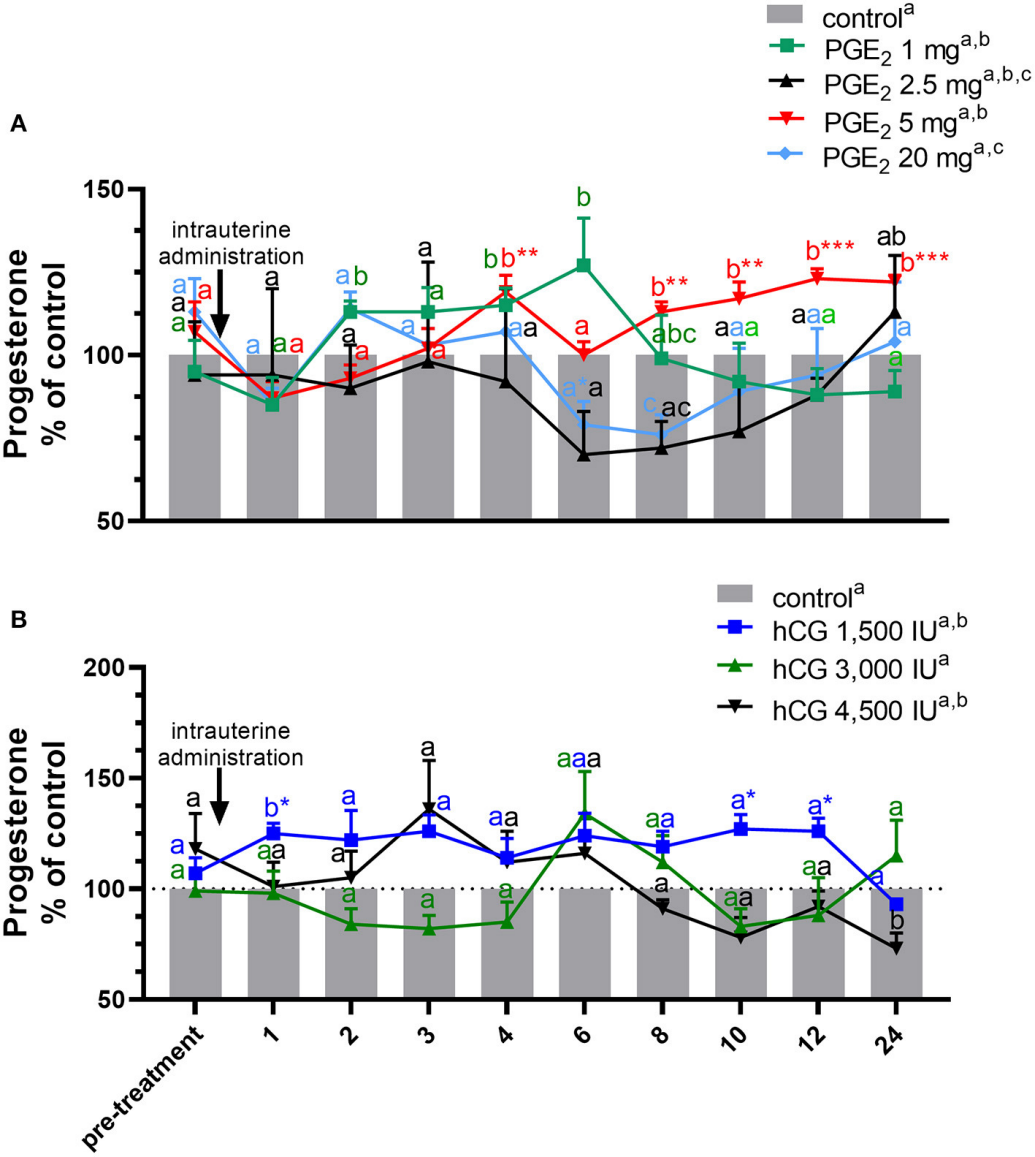
Concentration of progesterone (P_4_) in the jugular vein blood plasma in mares with one intrauterine administration of **(A)** four different doses of prostaglandin (PG) E_2_ (PGE_2_; 1, 2.5, 5, 20 mg/5 ml, *n* = 3/dose) and **(B)** three different doses of human chorionic gonadotropin (hCG; positive control; 1,500, 3,000, 4,500 IU/5 ml, *n* = 3/dose) on day 10 of the estrous cycle, compared with control groups. All values are presented as % of the control. Different superscript letters ^a, b, c^ indicate significant differences in P_4_ concentrations between PGE_2_- or hCG-treated group vs. control group at specific time points of blood sample collection. Asterisks indicate significant differences between P_4_ levels in PGE_2_- or hCG-treated group vs. average concentration of P_4_ in blood plasma in the period before treatment (pre-treatment time: −2 to 0 h). The results were considered significantly different at *p* < 0.05.

In experiment 1, the differences in P_4_ concentrations in blood plasma samples between groups treated with different doses of PGE_2_ or hCG and control group were measured as the area under the curve (AUC), using the total amount of P_4_ concentrations (mean ± SEM) secreted during the experiments (Table 1). The differences in concentrations of P_4_ (Figure 2), and total PGF_2α_ concentrations (Table 2) in blood plasma samples in response to treatment with different doses of PGE_2_ or hCG were analyzed using a repeated measures design approach in which treatments and time of sample collection (h) were fixed effects and all interactions were included (two-way ANOVA test followed by Dunnett's multiple comparison test).

**Table 1:** The effect of one intrauterine administration of prostaglandin (PG) E_2_ or human chorionic gonadotropin (hCG; positive control) on progesterone (P_4_) concentrations in mares' blood plasma samples (*n* = 3 per dose) at day 10 of the estrous cycle.

**Group**	**Dose**	**Progesterone**	
		**Baseline (ng/ml)**	**Total amount (mean ±SEM)**
Control	Saline	10.78	80.44 ± 5.12
PGE_2_	1 mg	10.31	79.31 ± 22.10
	2.5 mg	10.21	68.6 ± 37.52
	5 mg	11.59	115.00 ± 8.99^*^
	20 mg	12.29	38.36 ± 50.91
hCG (positive control)	1,500 IU	11.67	115.2 ± 10.17^*^
	3,000 IU	10.91	81.81 ± 52.74
	4,500 IU	12.91	31.76 ± 16.84

*All values are expressed as total amount of P_4_ secretion (area under curve). Baseline indicates average concentration of P_4_ (ng/ml) in the period before treatment (pre-treatment time: −2 to 0 h). Asterisks indicate significant differences in P_4_ concentrations in PGE_2_- or hCG-treated group versus control group. The results were considered significantly different at p < 0.05*.

**Table 2:** The effect of one intrauterine administration of prostaglandin (PG) E_2_ on total prostaglandin F_2α_ (the sum of PGF_2α_ and PGF_2α_ metabolite 13,14-dihydro-15-keto PGF_2α_–PGFM) concentrations in mares' blood plasma samples (*n* = 3 per dose) at day 10 of the estrous cycle.

**Time (h)**	**Total prostaglandin F_2α_ (pg/ml)**				
	**Intra-U administration**				
	**Saline (control)**	**1 mg PGE_2_**	**2.5 mg PGE_2_**	**5 mg PGE_2_**	**20 mg PGE_2_**
−2	67.37 ± 10.57^a^	73.90 ± 13.79^a^	56.10 ± 1.56^a^	70.43 ± 13.03^a^	65.58 ± 2.57^a^
−1	82.19 ± 11.06^a^	89.00 ± 11.48^a^	70.48 ± 1.28^a^	89.48 ± 16.55^a^	79.96 ± 3.11^a^
0	92.00 ± 10.95^a^	96.10 ± 12.75^a^	68.48 ± 1.47^a^	92.53 ± 11.21^a^	80.01 ± 4.49^a^
1	88.64 ± 4.10^a^	102.59 ± 20.81^a^	138.47 ± 4.77^**b**^	86.91 ± 7.11^a^	161.11 ± 9.80^**b**^
2	76.62 ± 9.17^a^	74.10 ± 16.01^a^	51.75 ± 4.05^a^	74.66 ± 9.13^a^	109.39 ± 2.54^**b**^
3	101.21 ± 12.30^a^	79.16 ± 15.88^a^	68.21 ± 0.03^a^	73.25 ± 3.49^a^	133.30 ± 2.37^**b**^
4	84.06 ± 10.82^a^	59.50 ± 6.79^a^	57.88 ± 1.88^a^	62.08 ± 3.51^a^	73.45 ± 15.62^a^
6	61.84 ± 1.72^a^	84.92 ± 21.59^a^	73.89 ± 7.98^a^	57.93 ± 5.57^a^	65.57 ± 11.33^a^
8	127.73 ± 18.13^a^	67.26 ± 10.59^a^	86.80 ± 4.19^a^	85.94 ± 10.57^a^	76.49 ± 5.73^a^
10	96.13 ± 9.48^a^	65.37 ± 22.81^a^	57.63 ± 7.52^a^	58.96 ± 2.61^a^	89.50 ± 1.14^a^
12	64.18 ± 5.39^a^	93.44 ± 14.36^a^	79.25 ± 15.41^a^	77.94 ± 16.17^a^	64.58 ± 12.66^a^
24	58.29 ± 5.94^a^	71.36 ± 18.76^a^	80.52 ± 26.61^a^	70.45 ± 4.88^a^	63.70 ± 6.16^a^

*All values are expressed as the mean ± SEM. Different superscript letters ^a, b^ within the column indicate significant differences in total PGF_2α_ concentrations in PGE_2_-treated group versus the period before treatment (pre-treatment time: −2 to 0 h). The results were considered significantly different at p <0.05*.

**Figure 3:**
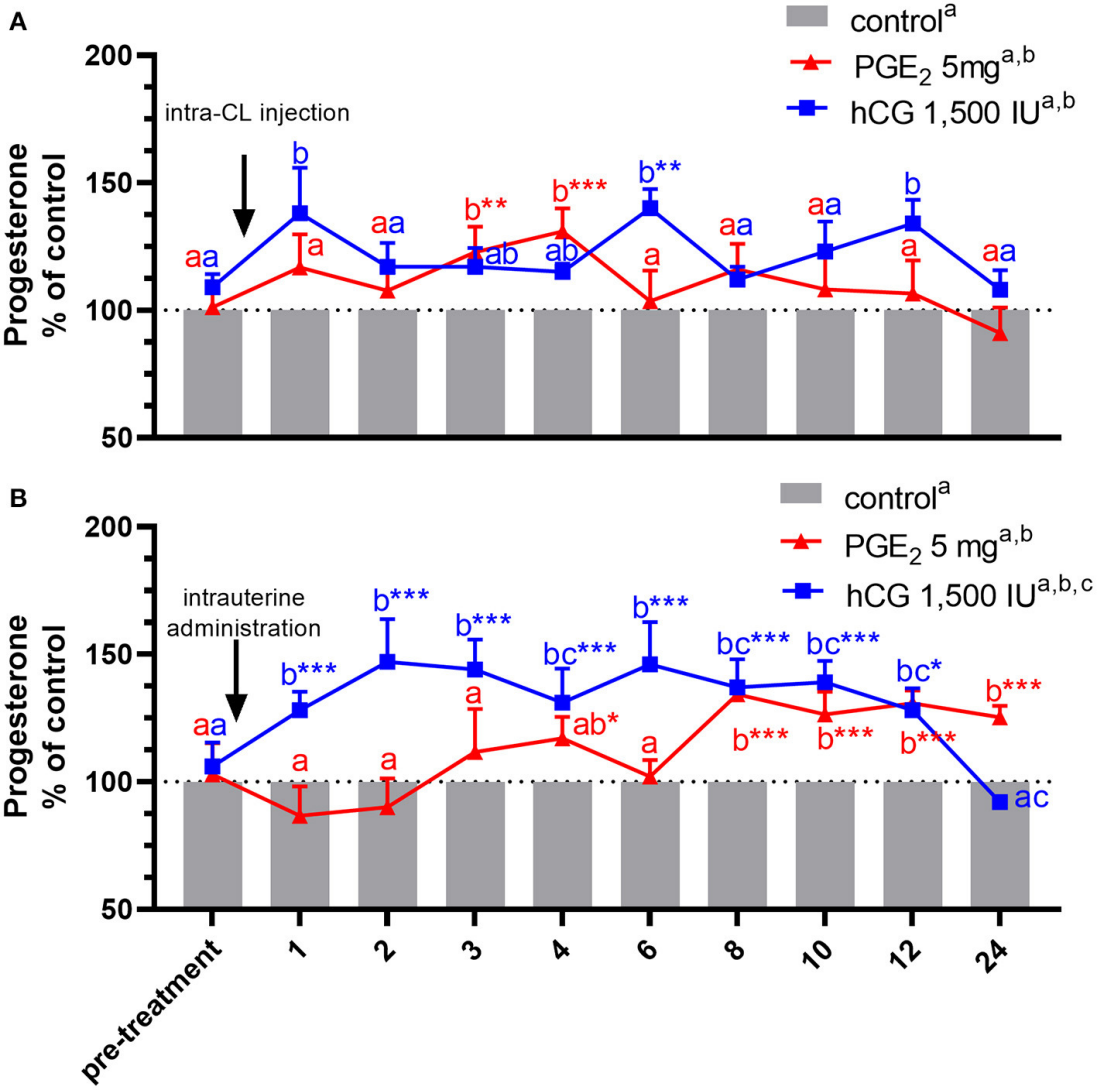
Concentrations of progesterone (P_4_) in the jugular vein blood plasma in mares with one **(A)** intra-CL injection of saline (control; gray bar), prostaglandin (PG) E_2_ (PGE_2_; 5 mg/ml; red line), or human chorionic gonadotropin (hCG, positive control; 1,500 IU/ml, blue line) or **(B)** one intrauterine administration of saline (control; gray bar), PGE_2_ (5 mg/5 ml; red line), or hCG (positive control; 1,500 IU/5 ml, blue line) on day 10 of the estrous cycle. All values were presented as % of the control. Different superscript letters ^a, b, c^ indicate significant differences between blood P_4_ level in PGE_2_- or hCG-treated groups of mares vs. control group at specific time points of blood sample collection. Asterisks indicate significant differences in blood P_4_ level within PGE_2_- or hCG-treated group of mares vs. average concentration of P_4_ in blood plasma in the period before treatment (pre-treatment time: −2 to 0 h). Average concentrations of P_4_ in the blood plasma samples of control mares during the period before treatment (pre-treatment time) were **(A)** 10.92 ng/ml or **(B)** 11.15 ng/ml, respectively. The results were considered significantly different at *p* < 0.05.

**Figure 4:**
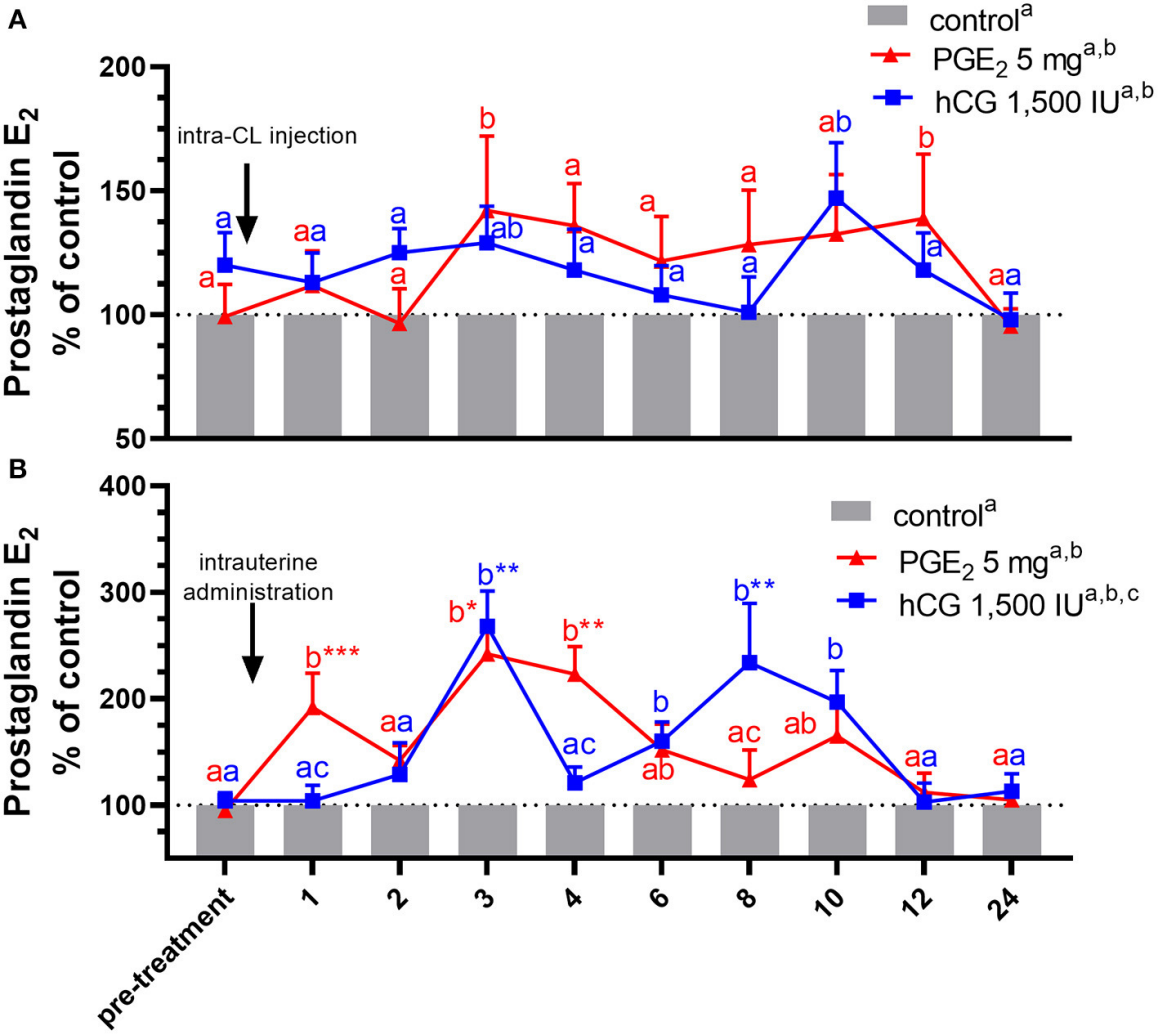
Concentrations of prostaglandin (PG) E_2_ in the jugular vein blood plasma in mares with one **(A)** intra-CL injection of saline (control; gray bar), PGE_2_ (5 mg/ml; red line), or human chorionic gonadotropin (hCG, positive control; 1,500 IU/ml, blue line) or **(B)** one intrauterine administration of saline (control; gray bar), PGE_2_ (5 mg/5 ml; red line), or hCG (positive control; 1,500 IU/5ml, blue line) on day 10 of the estrous cycle. All values are presented as % of the control. Different superscript letters ^a, b, c^ indicate significant differences between blood PGE_2_ level in PGE_2_- or hCG-treated groups of mares vs. control group at specific time points of blood sample collection. Asterisks indicate significant differences in blood PGE_2_ level within PGE_2_- or hCG-treated group of mares vs. average concentration of PGE_2_ in the period before treatment (pre-treatment time: −2 to 0 h). Average concentrations of PGE_2_ in the blood plasma samples of control mares during the period before treatment (pre-treatment time) were **(A)** 243.39 ng/ml or **(B)** 264.18, respectively. The results were considered significantly different at *p* < 0.05.

In experiment 2, data were analyzed by two-way ANOVA (treatments vs. the differences in P_4_ and PGE_2_ concentrations) (Figures 3, 4) and blood plasma samples collected after application of PGE_2_ or hCG were analyzed using a repeated measures design approach in which treatments and time of sample collection (h) were fixed effects and all interactions were included (two-way ANOVA test followed by Dunnett's multiple comparison test). All values are presented as percentage of the control. The differences in P_4_ (Table 3) and PGE_2_ (Table 4) concentrations in blood plasma samples between PGE_2_ or hCG groups were measured as AUC, using the total amount of P_4_ or PGE_2_ concentrations secreted during the experiments and were calculated using two-way ANOVA, followed by Dunnett's multiple comparisons test.

**Table 3:** The effect of one intra-CL injection or one intrauterine administration of prostaglandin (PG) E_2_ or human chorionic gonadotropin (hCG; positive control) on progesterone (P_4_) concentrations in mares' blood plasma samples (*n* = 6 per group) at day 10 of the estrous cycle.

**Group**	**Type of administration**	**Progesterone**
		**Total amount (mean ±SEM)**
PGE_2_	Intra-CL	13.55 ± 5.98^a^
5 mg	Intra-U	28.19 ± 6.05^ab^
hCG (positive control)	Intra-CL	23.25 ± 3.8^a^
1,500 IU	Intra-U	49.24 ± 7.47^b^

*All values are expressed as total amount of P_4_ secretion (area under curve). Different superscript letters ^a, b^ indicate significant differences in P_4_ concentrations between PGE_2_ and hCG groups. The results were considered significantly different at p <0.05*.

**Table 4:** The effect of one intra-CL injection or one intrauterine administration of prostaglandin (PG) E_2_ or human chorionic gonadotropin (hCG; positive control) on PGE_2_ concentrations in mares' blood plasma samples (*n* = 6 per group) at day 10 of the estrous cycle.

**Group**	**Type of administration**	**Prostaglandin E_2_**
		**Total amount (mean ±SEM)**
PGE_2_	Intra-CL	408.9 ± 205.9^a^
5 mg	Intra-U	994.8 ± 155.1^a^
hCG (positive control)	Intra-CL	457.9 ± 132.4^a^
1,500 IU	Intra-U	951.4 ± 176.7^a^

*All values are expressed as total amount of PGE_2_ secretion (area under curve). Different superscript letters ^a, b^ indicate significant differences in PGE_2_ concentration between PGE_2_ and hCG groups. The results were considered significantly different at p <0.05*.

## Results

### Experiment 1. Dose-Dependent Effect of Prostaglandin E_2_ on CL, Compared With Human Chorionic Gonadotropin Action

In mares, only one intra-U administration of PGE_2_ at the dose of 5 mg/5 ml increased the total amount of P_4_ concentrations in blood plasma, compared with the control group (*p* < 0.05; Table 1). An increase in P_4_ concentrations was observed at 4 h and between 8 and 24 h after intra-U administration of PGE_2_ at the dose of 5 mg/5 ml, compared with the control group and to its concentrations in the period before treatment (pre-treatment time) (*p* < 0.05; Figure 2A).

An increase in P_4_ concentrations in blood plasma was observed at 2 h and between 4 h and 6 h after intra-U administration of PGE_2_ at the dose of 1 mg/5 ml, compared with the control group (*p* < 0.05; Figure 2A), while intra-U administration of PGE_2_ at the dose of 20 mg/5 ml decreased its concentrations at 6 h compared with the pre-treatment time, and at 8 h, compared with the control group (*p* < 0.05; Figure 2A).

The total amount of P_4_ concentrations increased in blood plasma only after one intra-U administration of hCG at the dose of 1,500 IU/5 ml, compared with the control group (*p* < 0.05; Table 1). An increase in P_4_ concentrations was observed at 1 h after one intra-U administration of hCG at the dose of 1,500 IU/5 ml compared with the control group (*p* < 0.05; Figure 2B). Moreover, an increase in P_4_ concentrations was noticed at 1 h and between 10 and 12 h after intra-U hCG administration at the dose of 1,500 IU/5 ml, compared with its concentrations in the pre-treatment time (*p* < 0.05; Figure 2B).

An intra-U administration of hCG at the dose of 4,500 IU/5 ml decreased P_4_ concentrations at 24 h, compared with the control group (*p* < 0.001; Figure 2B).

Concentrations of PGF_2α_ and its metabolite PGFM (total PGF_2α_) in blood plasma samples increased at 1 h after an intra-U administration of PGE_2_ at the dose of 2.5 mg/5 ml, compared with its concentrations in the pre-treatment time (*p* < 0.05; Table 2). Moreover, an increase in total PGF_2α_ concentrations was observed between 1 and 3 h after an intra-U administration of PGE_2_ at the dose of 20 mg/5 ml, compared with its concentrations in the pre-treatment time (*p* < 0.05; Table 2).

### Experiment 2. Comparison of Intra-CL vs. Intra-U Application of Prostaglandin E_2_ on Corpus Luteum Function, Compared With Human Chorionic Gonadotropin Action

An increase in P_4_ concentrations in blood plasma samples was noticed in mares, between 3 and 4 h after receiving one intra-CL injection of PGE_2_, compared with its concentrations in the pre-treatment time within PGE_2_-treated group (*p* < 0.01), and with respect to the control mares (*p* < 0.01; Figure 3A). At the same time, P_4_ concentrations increased between 8 and 24 h after intra-U administration of PGE_2_, compared with P_4_ concentrations in the pre-treatment time within PGE_2_-treated group (*p* < 0.001), as well as compared with the control mares (*p* < 0.05; Figure 3B).

In mares, an intra-CL injection of hCG elevated P_4_ levels at 6 h, compared with P_4_ levels in the pre-treatment time within PGE_2_-treated group (*p* < 0.05; Figure 3A). Moreover, one intra-CL injection of hCG (positive control) increased P_4_ concentrations in blood plasma at 1, 6, and 12 h after its application, compared with the control group (*p* < 0.001; Figure 3A), while its intra-U administration elevated P_4_ concentrations between 1 and 12 h, compared with the control group, and to P_4_ concentrations in the pre-treatment time within this group of mares (*p* < 0.05; Figure 3B). Total amount of P_4_ found in mares with intra-U administration of hCG was greater compared with total amount of P_4_ in mares with its intra-CL injection (*p* < 0.05; Table 3).

In mares, an intra-CL injection of PGE_2_ increased PGE_2_ concentrations in blood plasma at 3 and 12 h after its administration, compared with the control group (*p* < 0.05; Figure 4A), while an intra-CL injection of hCG (positive control) increased its concentrations at 10 h after injection, compared with the control group (*p* < 0.05; Figure 4A). Prostaglandin E_2_ concentrations were elevated after intra-U administration of PGE_2_ at 1 h and between 3 and 4 h, relative to the control mares (*p* < 0.001), and to PGE_2_ levels in the pre-treatment time (*p* < 0.05; Figure 4B). Moreover, intra-U administration of hCG (positive control) increased PGE_2_ concentrations at 3 h and between 6 and 10 h after its administration, compared with the control group (*p* < 0.01; Figure 4B), and at 3 and 8 h after hCG administration, compared with PGE_2_ levels in the pre-treatment time (*p* < 0.01; Figure 4B). No differences in the total amount of PGE_2_ were observed between mares with intra-U administration and intra-CL injection (*p* > 0.05; Table 4).

## Discussion

Until now, many studies have been focusing on the different application route or sites of luteolytic/luteotropic factors that may be used in veterinary practices to regulate the estrous cycle in mares. In the literature, different ways of PGE_2_ or hCG administrations have been demonstrated, for example, i.m., i.v., s.c., intrafollicular, or intracervical (10, 14, 17, 18, 58, 59). The ultrasound-guided intra-CL injection as a method for studying the direct effect of PGF_2α_ on reproductive function in mares was evaluated by Weber et al. (61). However, this technique is not widely known by practitioners. While intra-U administration of luteotropic PGE_2_ on the CL function was described by Vanderwall et al. (32), in our study we demonstrated the effect of luteotropic factor PGE_2_ on P_4_ secretion, depending on the application site: intra-CL vs. intra-U in mares at day 10 of the estrous cycle. To the best of our knowledge, for the first time, we have showed that application of PGE_2_ supports equine CL secretory function, regardless of the application site, consequently leading to differences in both P_4_ and PGE_2_ concentrations in blood plasma.

The role of PGE_2_ on equine CL function is not fully understood. A previous *in vitro* study in cows confirmed that PGE_2_ participates in luteoprotective mechanisms required for CL formation and maintenance (62), and stimulates the P_4_ production by luteal steroidogenic cells (63). Moreover, in cows and ewes, there have been evidences that PGE_1_ or PGE_2_ prevented P_4_-induced premature luteolysis by suppressing the loss of luteal LH receptors (64, 65).

Interestingly, our study shows that the action of PGE_2_ on CL secretory function is determined by the application site and dose. An intra-CL injection of PGE_2_ increased P_4_ concentrations in blood plasma of mares at day 10 of the estrous cycle compared with the control group, suggesting its direct action. The aforementioned data are in agreement with a preliminary study conducted by our group (33, 34), showing that in mares PGE_2_ plays a luteotropic role as an auto-paracrine factor stimulating P_4_ production by luteal steroidogenic cells and CL tissues *in vitro*. Some decades ago, Vanderwall et al. (32) reported that a single intra-U administration of PGE_2_ was capable to maintain prolonged luteal function in the mare *in vivo*. In the experiment of Vanderwall et al. (32), non-pregnant mares were continuously infused with 0.24 mg of PGE_2_, from day 10 to 16 postestrus, using an osmotic minipump surgically placed into the uterine lumen. In our study, intra-U administration of PGE_2_ increased P_4_ concentrations in blood plasma on day 10 of the estrous cycle in mares, compared with the control group. Simple comparison between data obtained in our study and in the study of Vanderwall et al. (32) cannot be made because of differences in methodology of PGE_2_ application. We should take into account that in our study, whereas P_4_ concentrations increased at 3–4 h after direct intra-CL injection of PGE_2_, the positive effect of intra-U administration of PGE_2_ on P_4_ concentrations was observed between 8 and 24 h after treatment. We suppose that the aforementioned effect is a result of indirect action of PGE_2_ on PGE_2_ receptors in the uterus, involving the regulation of vasculature events and induction of other luteotropic factors engaged in luteal support, in the equine endometrium (e.g., growth factors, nitric oxide, and cytokines). Galvão et al. (42, 66) showed that cytokines interact with nitic oxide synthases and influence luteal angiogenesis in mares as angiogenic factors themselves can also modulate luteal secretory function. Previously, Otzen et al. (67) found that PGE_2_ stimulates vascular endothelial growth factor (VEGF), which participates in the regeneration and expansion of the equine uterine blood vessel network. Moreover, VEGF has been reported to effectively modulate luteal secretory function of equine CL (P_4_ and PGE_2_ production) (66).

In the first experiment, the dose of PGE_2_ 5 mg/5 ml was chosen as an effective dose based on an increase in P_4_ concentrations in blood samples after intra-U treatments in mares. We demonstrated that the highest dose of PGE_2_ administered into the uterus does not affect P_4_ concentrations in blood plasma. Therefore, we can suspect the possibility of the conversion of PGE_2_ by the PGE2-9-K into PGF_2α_. It is known that PGE2-9-K enzyme has also a 20 α-HSD activity, and in fact converts P_4_ into 20α-OH-P_4_, which may contribute to the decrease of P_4_ induced by PGF_2α_ (4, 48). To check and confirm this fact, we examined the effect of an intra-U administration of different doses of PGE_2_ on total PGF_2α_ (sum of PGF_2α_ and its main metabolite—PGFM) concentrations in blood plasma on day 10 of the estrous cycle in mares. Interestingly, we observed higher total PGF_2α_ concentrations in blood plasma between 1 and 3 h after intra-U administration of PGE_2_ at the highest dose (20 mg/5 ml), compared with its concentrations in the pre-treatment time. Hence, our *in vivo* results should be interpreted carefully and our hypothesis that the lack of the effect of PGE_2_ in the highest dose on P_4_ concentrations may be related to its conversion into PGF_2α_ by PGE2-9-K needs further studies in mares.

In our study, we assume that intra-CL injection and intra-U administration of PGE_2_ increased its own concentration in blood plasma. There is evidence that in the endometrium of mare, PGF_2α_ has an auto-amplification system, stimulating its own production (40). Therefore, future study should be planned to assume whether there is a positive PGE_2_ feedback loop and whether PGE_2_ has a positive effect on its own production.

There are a large number of *in vivo* studies concerning the effect of hCG on CL function in mare (16–19, 68). Kelly et al. (18) and Watson et al. (19) demonstrated the positive luteotropic effect of hCG on P_4_ secretion. Therefore, in our study, we decided to assign mares treated with intra-CL injection or intra-U administration of hCG as positive control group. In the present study, we observed an increase in P_4_ concentration in blood plasma after intra-U administration of 1,500 IU of hCG. No effect on CL function was reported by Brito et al. (68), using one i.v. injection of this same dose-−1,500 IU of hCG at day 10 after ovulation. In agreement with our results, a positive effect on P_4_ secretion was observed in diestrus mares, using repeated i.m. injections of 1,000 IU of hCG (days 3, 4, 5) (18) or a single i.v. injection of 1,500 IU of hCG (day 8) (19). Interestingly, in our study one intra-U administration of hCG at the doses 3,000 IU or 4,500 IU did not affect P_4_ secretion from equine CL. Likewise, Köhne et al. (16) did not observe any increase in P_4_ concentration and luteal size after i.v. administration of 5,000 IU of hCG at day 5 after ovulation. Therefore, it might be suggested that higher doses of hCG are not related to their effectiveness. We have noted that both a single intra-CL injection of hCG and a single intra-U administration of hCG increased blood P_4_ concentrations, supporting P_4_ secretion from mare CL. The intra-CL injection of hCG seems to directly influence the luteal steroidogenic cells. An additional *in vitro* study should be conducted to explore molecular mechanisms involved in the CL secretory function in response to intra-CL injection of hCG. Unexpectedly, the intra-U administration of hCG was more effective in increasing P_4_ secretion by CL (Table 3), throughout its indirect effect on equine PGE_2_ receptors in the uterus, affecting regulation of vasculature events and induction of luteotropic factors involved in luteal support.

Human chorionic gonadotropin has structural and functional similarities with LH, sharing the same receptor with this luteotropic hormone (1). The evidence for the presence of the LH/CGR receptor in the reproductive tract of humans and other domestic animals is well described (11, 12). In mares, LH receptor expression occurs in the CL (69) and in the endometrium and myometrium during the estrous cycle and anestrus (13). Therefore, the presence of LH/CGR receptors in equine reproductive tract could mediate the indirect effect of intra-U administration of hCG and the direct effect of hCG injection into the CL. Interestingly, in the present study, we show that only one intra-U administration of hCG increases PGE_2_ concentration in blood plasma. We have previously demonstrated that LH stimulated PGE_2_ secretion by equine endometrium and myometrium (45). We postulate that hCG through LH/CGR receptors in the mare uterus affects the luteotropic PGE_2_ production. Moreover, PGE_2_ has a positive effect on P_4_ secretion. However, further studies are needed to clarify the mechanism of action of hCG on PGE_2_ production within the equine reproductive tract.

In conclusion, the aforementioned results indicate the importance of proper application site of drugs and may influence drug delivery strategies in veterinary medicine. Application of PGE_2_ supports equine CL function via augmentation of P_4_ and PGE_2_ secretions. Progesterone secretion in response to PGE_2_ depends on their application site. In the present study, we found more effective increase in P_4_ secretion after intra-U administration of luteotropic factors (especially hCG) than their intra-CL injections. Therefore, the efficacy of intra-CL site of application warrants further *in vitro* and *in vivo* studies. We confirm that therapeutic use of intra-U administration of luteotropic factors is an easily applicable, valuable method in veterinary practice that may be used to support early pregnancy in mares. However, this knowledge is still insufficient and needs better understanding of the endocrine, cellular, receptor, and molecular mechanism action of luteotropic factors on equine CL function.

## Data Availability Statement

The raw data supporting the conclusions of this article will be made available by the authors, without undue reservation.

## Ethics Statement

The animal study was reviewed and approved by Local Ethics Committee for Experiments on Animals, University of Warmia and Mazury in Olsztyn, Poland (Approval No. 51/2011). Written informed consent was obtained from the owners for the participation of their animals in this study.

## Author Contributions

KKP-T: conceptualization, investigation, methodology, formal analysis, visualization, writing—original draft, and writing—review and editing. AWJ: investigation, methodology, formal analysis, visualization, writing—original draft, and writing—review and editing. AZS-M: conceptualization, investigation, methodology, formal analysis, writing—original draft, and writing—review and editing. EŻ: formal analysis. GF-D: supervision and writing—review and editing. DJS: conceptualization, investigation, formal analysis, supervision, funding acquisition, and writing—review and editing. All authors have read, critically revised, and approved the final version of the article.

## Funding

This work was supported by the National Science Center in Poland (2011/02/A/NZ5/00338).

## Conflict of Interest

The authors declare that the research was conducted in the absence of any commercial or financial relationships that could be construed as a potential conflict of interest.

## Publisher's Note

All claims expressed in this article are solely those of the authors and do not necessarily represent those of their affiliated organizations, or those of the publisher, the editors and the reviewers. Any product that may be evaluated in this article, or claim that may be made by its manufacturer, is not guaranteed or endorsed by the publisher.
