# Supplementary material for: The Effects of Prostaglandin E2 Treatment on the Secretory Function of Mare Corpus Luteum Depends on the Site of Application: An in vivo Study
Source: Front Vet Sci. 2022 Feb 15;8:753796. doi: 10.3389/fvets.2021.753796 (PMC8885592; doi:10.3389/fvets.2021.753796)
Supplement: Supplementary file 1 [file Data_Sheet_1.docx]

Supplementary Material

# Supplementary Tables

|  | Treatments | Time | Interaction |
| --- | --- | --- | --- |
| Progesterone | 4.27^**^ | 1.355 | 1.161 |

**Supplementary Table 1.** The effect of one intrauterine administration of prostaglandin (PG) E_2_ on progesterone (P_4_) concentration in blood plasma samples of mares. Two-way ANOVA F values: treatments (intra-U administration of: saline, PGE_2_) vs. time (pre-treatment, 1-, 2-, 3-, 4-, 6-, 8- 10-, 12- 24 h). *DF*: treatments, 4; time, 9; interaction, 36; ^*^*P* <0.01

|  | Treatments | Time | Interaction |
| --- | --- | --- | --- |
| Progesterone | 0.708 | 14.39^***^ | 5.513^***^ |

**Supplementary Table 2.** The effect of one intrauterine administration of human chorionic gonadotropin (hCG) on progesterone (P_4_) concentration in blood plasma samples of mares. Two-way ANOVA F values: treatments (intra-U administration of: saline, hCG) *vs.* time (pre-treatment, 1-, 2-, 3-, 4-, 6-, 8- 10-, 12- 24 h). *DF*: treatments, 3; time, 9; interaction, 33; ^*^*P* <0.001

|  | Treatments | Time | Interaction |
| --- | --- | --- | --- |
| Total PGF_2α_ (PGF_2α_ and its main metabolite PGFM) | 2.677 | 8.688^***^ | 2.739^***^ |

**Supplementary Table 3**. The effect of one intrauterine administration of prostaglandin (PG) E_2_ on total PGF_2α_ concentration in blood plasma samples of mares. Two-way ANOVA *F* values: treatments (intra-U administration of: saline, PGE_2_) *vs.* time (pre-treatment, 1-, 2-, 3-, 4-, 6-, 8- 10-, 12- 24 h). *DF*: treatments, 4; time, 9; interaction, 36; ^*^*P* <0.05

|  | Treatments | Time | Interaction |
| --- | --- | --- | --- |
| Progesterone | 5.182^*^ | 6.166^***^ | 2.2852^***^ |
| Prostaglandin E_2_ | 2.098 | 1.522 | 1.208 |

**Supplementary Table 4**. The effect of one intrauterine administration of prostaglandin (PG) E_2_ or human chorionic gonadotropin (hCG) on progesterone (P_4_) concentration in blood plasma samples of mares. Two-way ANOVA *F* values: treatments (intra- CL injection of: saline, PGE_2_, hCG) *vs.* time (pre-treatment, 1-, 2-, 3-, 4-, 6-, 8- 10-, 12- 24 h). *DF*: treatments, 2; time, 9; interaction, 18; ^*^*P* <0.05

|  | Treatments | Time | Interaction |
| --- | --- | --- | --- |
| Progesterone | 9.689^**^ | 14.95^***^ | 4.711^***^ |
| Prostaglandin E_2_ | 17.63^***^ | 5.214^***^ | 5.772^***^ |

**Supplementary Table 5**. The effect of one intrauterine administration of prostaglandin (PG) E_2_ or human chorionic gonadotropin (hCG) on PGE_2_ concentration. Two-way ANOVA *F* values: treatments (intra- U administration of: saline, PGE_2_, hCG) *vs.* time (pre-treatment, 1-, 2-, 3-, 4-, 6-, 8- 10-, 12- 24 h). *DF*: treatment, 2; time, 9; interaction, 18; ^*^*P* <0.01
